# Gastric Linitis Plastica and Peritoneal Carcinomatosis as First Manifestations of Occult Breast Carcinoma: A Case Report and Literature Review

**DOI:** 10.1155/2018/4714708

**Published:** 2018-07-08

**Authors:** Mara Mantiero, Giovanni Faggioni, Alice Menichetti, Matteo Fassan, Valentina Guarneri, Pierfranco Conte

**Affiliations:** ^1^Medical Oncology Unit 2, Istituto Oncologico Veneto, IRCCS, Padova, Italy; ^2^Department of Surgical, Oncological and Gastroenterological Sciences, University of Padova, Padova, Italy; ^3^Department of Medicine, Surgical Pathology & Cytopathology Unit, University of Padova, Padova, Italy

## Abstract

Gastric linitis plastica is a diffuse involvement of the stomach walls by neoplastic cells. It represents about 3–19% of primitive gastric adenocarcinomas, but it can also be the manifestation of a metastatic disease. Breast cancer is the most frequent malignancy in women, and the metastatic spread to the stomach occurs in less than 10% of the cases. We present an unusual case of gastric linitis plastica and peritoneal carcinomatosis as manifestations of an occult breast cancer in a 53-year-old woman. Imaging and endoscopic evaluation were not able to discriminate a primary from a secondary gastric lesion. The histological evaluation excluded the diagnosis of a primary gastric neoplasia. The IHC profile was consistent with the diagnosis of metastases from the breast cancer. Due to the hormonal receptors' positivity, we started therapy with fulvestrant (500 mg, day 0, 14, and 28 and every 28 days thereafter by intramuscular injection). After 20 months, the same therapy is still ongoing and well tolerated, while the patient is in good condition with improvement of the dysphagia. Almost 2 years after the diagnosis of linitis plastica, the primitive breast lesion is still occult.

## 1. Introduction

Metastatic cancer of unknown primary (CUP site syndrome) is characterized by the presence of the metastatic lesion without the primitive carcinoma. It accounts for 3–5% of all solid malignant tumours, and the prognosis is generally poor [1]. Only microscopic analysis, with histological and immunohistochemical exam, can define the primary origin of the lesion, and it is fundamental for the clinician to define the correct treatment plan. The discussion with the pathologist is essential.

Metastasis from breast cancer to the gastrointestinal tract is rare, less than 10% [2], and typically occurs many years after the diagnosis.

We present an unusual case of gastric linitis plastica and peritoneal carcinomatosis as first manifestations of an occult breast cancer. The correct identification of the primary origin of the lesion was crucial to avoid a potentially useless gastric surgery.

## 2. Case Presentation

In March 2016, a 53-year-old premenopausal woman was admitted to our institute with the diagnosis of gastric linitis plastica and peritoneal carcinomatosis. She presented with upper abdominal pain, dyspepsia, nausea, and daily postprandial vomiting with weight loss of approximately 4 kilograms in 2 months. The Eastern Cooperative Oncology Group (ECOG) performance status (PS) was 2. Her medical history was negative for oncologic diseases, and she had no relevant comorbidities; no history of *Helicobacter pylori*-associated gastritis. At clinical examination, she presented with epigastric tenderness and no mass. Blood tests were within the normal values, with the exception of CA15.3 (211 U/ml) and CEA (11.1 ng/ml). Abdominal computed tomography (CT) revealed an increased wall thickness of the pyloric antrum along with mesenteric lymphadenopathy (20 mm) and peritoneal carcinomatosis. No liver metastases were detected. At esophagogastroduodenoscopy (EGDS), a severe pyloric stenosis was reported in the absence of mucosal lesions. The clinical manifestation was strongly suggestive of linitis plastica. Several gastric biopsies were performed, and histology concluded for a diffuse localization of epithelial cancer. Immunohistochemistry excluded gastrointestinal origin. There was a strong immunoreactivity for estrogen and progesterone receptors (ER-PgR: 80%-80%), GATA3 (3+), and cytokeratin (CK) 7, 8, 18, and 19; the human epithelial growth factor receptor 2 (HER2) was negative (1+) and the Ki67 index was <5%. Histological exam concluded for metastatic breast cancer with gastric linitis plastica.

A complete breast radiological investigation including bilateral ultrasound and mammography, and magnetic resonance imaging excluded the presence of breast abnormalities. Multiple bilateral suspicious axillary lymph nodes (maximum diameter of approximately 10 mm) were identified at ultrasonography and MRI. A fine-needle aspiration of a right axillary lymph node was performed, and cytology was positive for epithelial malignant cells.

To definitively exclude a gastrointestinal origin of the neoplasm, the patient also underwent laparoscopic peritoneal biopsy. Histological and immunohistochemical studies confirmed breast origin. After the multidisciplinary discussion, a surgical approach was excluded. A Witzel feeding jejunostomy was created.

All international breast cancer guidelines recommend endocrine therapy in luminal metastatic breast cancer without visceral crisis. Our patient, after jejunostomy creation and starting of enteral nutrition, was asymptomatic, and so, in April 2016, hormone therapy with fulvestrant was started (500 mg, day 0, 14, and 28 and every 28 days thereafter by intramuscular injection). We decided on intramuscular therapy to overcome the patient's dysphagia.

After four months of hormone therapy, CT scan was performed and reported stable disease. The patient also experienced clinical improvement with weight increase (1 kg) and palliation of dysphagia. Sporadic postprandial vomiting was still present.

**Figure 1:**
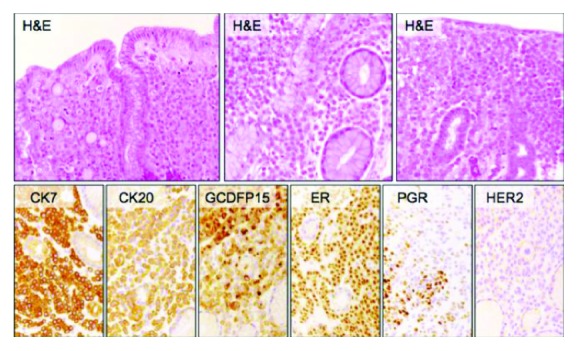
Histology confirmed localization of adenocarcinoma with immunohistochemistry: ER 90%, PgR 35%, CK7 3+, GCDFP-15 3+, and HER2 1+.

In January 2017, CA15.3 was normalized (3.8 U/ml) and a new EGDS with biopsies was performed. Histology confirmed localization of adenocarcinoma with immunohistochemistry ER 90%, PgR 35%, CK7 3+, gross cystic disease fluid protein 15 (GCDFP-15) 3+, and HER2 1+ (Figure 1).

The patient is still in a good clinical condition with ECOG PS 1 up to this day. Supportive enteral nutrition is still ongoing, but dysphagia has significantly improved. Hormone therapy with fulvestrant is still ongoing and well tolerated. The last radiological evaluation was performed in February 2018, and it showed a stable disease.

Additionally, because of a potential genetic correlation between diffuse gastric carcinoma and early-onset lobular breast carcinoma [3], we also performed a genetic evaluation and searched for CDH1 germline mutations, but no genetic abnormalities were identified. In our case, the absence of primitive lesion prevented any possibility of the histological subdefinition, although the lobular histological subtype is the most common cause of metastatic gastric linitis plastica caused by breast cancer [4].

## 3. Discussion

Breast cancer is the most common malignancy in women, accounting for about 30% of new diagnosis. Approximately 6–10% of new breast cancer cases are initially metastatic, and the most common sites of metastatization are the liver, lung, brain, and bone [5]. Metastases from breast cancer to the gastrointestinal tract are rare. Harris et al. published in 1984 the data about an autopsy series of 109 patients who died from breast cancer: 84% of them were metastatic and only 8.8% had gastric involvement [2].

Typically, metastatic spread to the gastrointestinal tract occurs many years after the diagnosis of breast cancer. In our case, it was at the onset of the disease. Gastric metastatization can have two different patterns of manifestation: nodular pattern with ulcerative masses, typical of invasive ductal carcinoma (IDC), or a diffuse mural involvement, typical of invasive lobular carcinoma (ILC). In the latter case, multiple and deep biopsies are recommended for the diagnosis because sometimes the scirrhous and fibrotic reaction can invade the gastric wall without mucosal involvement.

Although the cases described are not many, the lobular histological subtype is the most common cause of metastatic gastric linitis plastica caused by breast cancer [4]. Taal et al. performed a retrospective analysis in a 15-year period showing that 83% of patients with breast cancer and gastric metastasis have lobular histological subtype [6]. Rare cases of linitis plastica of the rectum as a possible clinical presentation of lobular breast carcinoma are also described [7–10]. However, the biological mechanism underlying this unusual correlation is not yet clear.

The presence of the metastatic lesion without primitive carcinoma represents a heterogeneous group defined as “carcinoma of unknown primary” (CUP). They account for 3–5% of all tumors, and the prognosis is poor [1]. Probably, these tumors acquire the capacity to metastasize before the development of a clinically evident primary lesion [11]. A historical autopsy study showed that the breast was the primary tumor site in CUP syndrome in only 2% of the cases [12, 13].

Immunohistochemistry is fundamental to correctly identify the primary site and, in our case, was essential to decide the therapeutic strategy. Since about 80% of human breast cancer cells express hormone receptors, ER and PR statuses are usually used as reliable markers for breast origin [14]. However, the primary gastric carcinomas can also express sex hormone receptors. According to Tokunaga and colleagues, the rates of positivity are about 26.6% for ER and 20.6% for PR [15]. In a more recent analysis by Matsui et al., the positivity is about 32% and 12% for ER and PR, respectively [16]. For this reason, their use, in association with other supplemental diagnostic markers, can improve the diagnostic accuracy. From an IHC point of view, breast cancer is positive for CK7 and CK18 and negative for CK20, as our patient. CK7 and CK20 are the first steps in the IHC markers' approach used in CUP syndrome. Cytoplasmatic positivity for GCDFP-15 is also highly specific (90%) to identify a malignant breast lesion. GCDFP-15 is a marker of apocrine differentiation and is detected in 62–72% of breast cancers [17, 18].

Probably in the future, the RNA microarray with gene expression tests will play an important role in the diagnosis of CUP. Su et al. defines a predictive algorithm using 110 genes expressed in the 11 most frequent malignancies. In their study, they have been able to predict the anatomical site of the tumor origin for 90% of the 175 carcinomas analyzed, including 9 of the 12 metastatic lesions [19]. The role of RNA profiling is evolving. More studies are ongoing, but the available data are still premature. More studies are needed to understand if gene expression can be different between primary and metastatic lesions.

The management of metastatic linitis plastica of the stomach is totally different from that of primary gastric carcinoma. Surgical resection is the first option for patients with primary gastric cancer without metastasis, but, in our case, gastric lesion was the manifestation of a systemic disease. All international breast cancer guidelines recommend endocrine therapy in luminal metastatic breast cancer without visceral crisis. For this reason, after the resolution of the symptoms with the jejunostomy creation, we decided to start systemic therapy with fulvestrant.

In conclusion, interaction between clinician and pathologist is important to select the correct IHC tests to perform.

Our goal in this case report is twofold: firstly, to improve the knowledge of surgeons and clinicians reminding them the need to rule out the possibility of a breast origin in women with gastric involvement, even in patients without a previous or concurrent history of breast carcinoma; secondly to increase the attention on immunohistochemical analysis.

To our knowledge, our case is the first published paper on CUP syndrome of breast cancer with this peculiar type of presentation. This case could be helpful with other clinicians due to its rarity and its unusual outcome.

## Abbreviations

CUP: Carcinoma of unknown primary.
